# Implication of nucleotides near the 3′ end of 16S rRNA in guarding the translational reading frame

**DOI:** 10.1093/nar/gkae143

**Published:** 2024-03-07

**Authors:** Alexandria Smart, Laura Lancaster, John Paul Donohue, Dustin Niblett, Harry F Noller

**Affiliations:** Center for Molecular Biology of RNA and Department of Molecular, Cell and Developmental Biology, University of California at Santa Cruz, Santa Cruz, CA, USA; Center for Molecular Biology of RNA and Department of Molecular, Cell and Developmental Biology, University of California at Santa Cruz, Santa Cruz, CA, USA; Center for Molecular Biology of RNA and Department of Molecular, Cell and Developmental Biology, University of California at Santa Cruz, Santa Cruz, CA, USA; Center for Molecular Biology of RNA and Department of Molecular, Cell and Developmental Biology, University of California at Santa Cruz, Santa Cruz, CA, USA; Center for Molecular Biology of RNA and Department of Molecular, Cell and Developmental Biology, University of California at Santa Cruz, Santa Cruz, CA, USA

## Abstract

Loss of the translational reading frame leads to misincorporation and premature termination, which can have lethal consequences. Based on structural evidence that A1503 of 16S rRNA intercalates between specific mRNA bases, we tested the possibility that it plays a role in maintenance of the reading frame by constructing ribosomes with an abasic nucleotide at position 1503. This was done by specific cleavage of 16S rRNA at position 1493 using the colicin E3 endonuclease and replacing the resulting 3′-terminal 49mer fragment with a synthetic oligonucleotide containing the abasic site using a novel splinted RNA ligation method. Ribosomes reconstituted from the abasic 1503 16S rRNA were highly active in protein synthesis but showed elevated levels of spontaneous frameshifting into the -1 reading frame. We then asked whether the residual frameshifting persisting in control ribosomes containing an intact A1503 is due to the absence of the N6-dimethyladenosine modifications at positions 1518 and 1519. Indeed, this frameshifting was rescued by site-specific methylation *in vitro* by the ksgA methylase. These findings thus implicate two different sites near the 3′ end of 16S rRNA in maintenance of the translational reading frame, providing yet another example of a functional role for ribosomal RNA in protein synthesis.

## Graphical Abstract

**Graphical Abstract:**
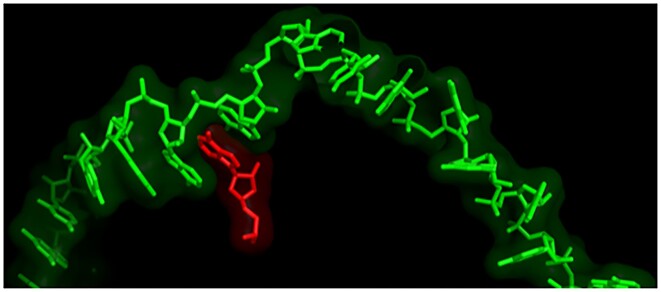

## Introduction

Accurate maintenance of the translational reading frame is a crucial cellular function; shifting to the +1 or –1 frame leads to miscoding and premature termination of the polypeptide chain (1), which can have lethal consequences. During translocation of mRNA and tRNA through the ribosome, tRNA movement is coupled to rotation of the head domain of the small ribosomal subunit (2–4). However, mRNA movement is presumably passive, dependent on its connections to tRNA through the inherently weak base pairing between codon and anticodon. During translocation, these codon-anticodon duplexes must leave the stabilizing environments of the ribosomal A and P sites and thus become vulnerable to frameshifting. During movement from the A site to the P site, codon-anticodon pairing is stabilized by contact between the tip of domain 4 of elongation factor EF-G with the minor groove surface of the duplex (2,4–6). Accordingly, in the absence of EF-G, spontaneous translocation leads to a stalled intermediate state showing a shift to the –1 reading frame (7). However, little is known about the potential contributions of the ribosome itself to maintenance of the reading frame during translocation. Here, we present evidence for the participation of 16S ribosomal RNA in this function.

**Figure 1.**
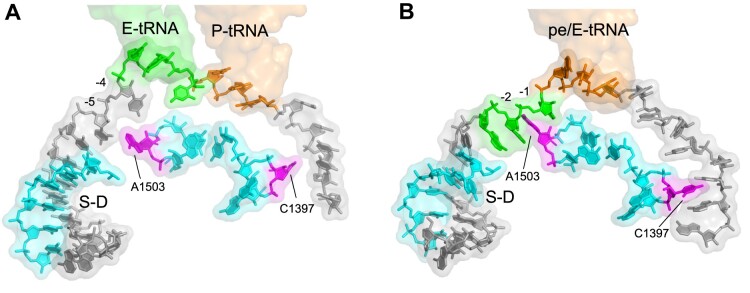
Intercalation of A1503 Between mRNA Bases. (**A**) A1503 in its retracted position in a classical-state ribosome complex (PDB code 4V6F) (8). (**B**) Intercalation of A1503 between bases –1 and –2 of the mRNA in ribosomes trapped in a chimeric-hybrid intermediate state of translocation (PDB code 4V9K) (3). Stacking of C1397 on position +10 of mRNA in the chimeric hybrid state is also shown. The positions of E-tRNA, P-tRNA and pe/E-tRNA are shown in transparent surface rendering. S–D, Shine–Dalgarno helix.

The crystal structure of a ribosome translocation complex trapped in an intermediate chimeric-hybrid state (3) revealed the unexpected intercalation of 16S rRNA base A1503 between bases –1 and –2 of the mRNA (where the first base of the P-site codon is defined as position +1; Figure 1). Inspection of subsequently published high-resolution structures shows that this phenomenon is widespread among certain translocation complexes, as described below. This observation raised the possibility that intercalation of A1503 might serve the purpose of preventing uncoupled movement of the mRNA and consequent shifting of the translational reading frame.

Since all four bases are capable of intercalation, we reasoned that base substitution mutations of A1503 would present an inadequate test of this possibility, we sought to eliminate intercalation of A1503 entirely by creating an abasic nucleotide at this position. Because of the infeasibility of chemical synthesis of full-length 16S rRNA, we introduced the abasic nucleotide by replacing the 3′-terminal 49-mer fragment (containing nucleotides 1494–1542) obtained by cleavage with the colicin E3 endonuclease (9) with a synthetic 49-mer, using a novel splinted RNA ligation method. The resulting semi-synthetic 16S rRNA was then reconstituted *in vitro* with ribosomal proteins and natural 50S subunits to produce 70S ribosomes. Using an *in vitro* assay, we tested the abasic ribosomes for activity in protein synthesis and for their ability to maintain the translational reading frame. Since these ribosomes also lacked the universally conserved *N*^6^,*N*^6^-dimethyladenosine modifications at positions 1518 and 1519 we also tested their effect on frameshifting. We found that both the abasic ribosomes and non-methylated ribosomes showed elevated levels of frameshifting, implicating two different sites near the 3′ end of 16S rRNA in maintenance of the translational reading frame.

## Materials and methods

### Structure analysis

Analysis of potential intercalation of 16S rRNA nucleotide A1503 was carried out on high-resolution X-ray and cryo-EM structures of 70S ribosome complexes that contain mRNA and one or more tRNAs, from the PDB and EMDB databases. Screening of 672 deposited ribosome structures identified a total of 209 in which the relative positions of A1503 and nearby mRNA bases could be placed with reasonable confidence, based on local resolution and/or map quality. We first used a computational approach to measure the positions of base A1503 relative to bases in the mRNA according to the distance between the centroids of the A1503 base and the nearest proximal mRNA bases. This was followed by visual inspection to identify structures in which A1503 is intercalated between two mRNA bases or stacked on a single mRNA base. Our screen identified 73 structures in which A1503 was intercalated, an additional 89 where it was stacked, and 47 structures where it was retracted out of range of intercalation or stacking.

Ribosome structures were assigned to specific conformational states according to the magnitudes of their intersubunit or 30S head rotation angles, as classical state (non-rotated), hybrid state (6°–10° 30S body rotation) or chimeric-hybrid state (15°–21° 30S head rotation) using the Euler-Rodrigues method, as previously described (11).

### Preparation of ribosomes and translation components

Tight-couple 70S ribosomes were isolated from *Escherichia coli* MRE600 as described (31–33). S100 extract was prepared from *E. coli* MRE600 cells and purified over DEAE resin as previously described (34). Total tRNA from *E. coli* was obtained from Sigma. Initiation factors IF1, IF2 and IF3 were overexpressed and purified as described (35). Initiator fMet-tRNA was prepared by *in vitro* transcription and cleavage with a *cis* HDV ribozyme present downstream from the tRNA sequence; the tRNA product was treated with T4 polynucleotide kinase to remove the resulting 2′,3′-cyclic phosphate, charged with methionine and acetylated, as described by Lancaster *et al.* (6).

### Construction of ribosomes containing an abasic nucleotide at position 1503 of 16S rRNA

Colicin E3 endonuclease was expressed from plasmid pDW1 (36), which encodes 6His-tagged Colicin E3 immunity protein and Colicin E3 endonuclease in tandem, and was purified essentially as described (36). The endonuclease was used to cleave the 16S rRNA in *E. coli* MRE600 70S ribosomes (37). The resulting long 16S rRNA fragment obtained from specific cleavage at position 1493 was purified by phenol extraction and sucrose gradient sedimentation. It was then ligated to a synthetic 49mer containing an abasic nucleotide at the position corresponding to A1503 of 16S rRNA (Dharmacon) with the following sequence (where the abasic nucleotide is shown in boldface and underlined):

5′-GUCGUAACA**A**GGUAACCGUAGGGGAACCUGCGGUUGGAUCACCUCCUUA-3′

Ligation was carried out with T4 RNA ligase 2 (38) using a tailed DNA splint that could be removed by annealing a complementary DNA oligonucleotide under conditions that preserve the higher-order structure of the rRNA. The DNA splint contained a core sequence complementary to the ends of the 1493mer and 49mer RNAs, flanked by non-complementary 5′ and 3′ tails. It could be removed efficiently at 46° by annealing to a competing DNA oligonucleotide with full complementarity to the entire tailed splint. A full description of the ligation procedure will be described elsewhere (Smart *et al.*, in preparation). For reconstitution of 30S subunits, 20 μM 16S rRNA was incubated with 25 μM total 30S proteins in a buffer containing 25 mM Tris–HCl (pH 7.5), 400 mM NH_4_Cl, 20 mM MgCl_2_ and 5 mM β-mercaptoethanol at 42° for 1 h. For reconstitution of complete 70S ribosomes, we added an equimolar amount of natural 50S subunits from *E. coli* MRE600 in a buffer containing 25 mM Tris–HCl (pH 7.5), 100 mM NH_4_Cl, 20 mM MgCl_2_ and 5 mM β-mercaptoethanol at 37° for 15 min. The resulting abasic ribosomes were purified by sedimentation over a 10–35% sucrose gradient in 25 mM Tris·HCl (pH 7.5), 100 mM NH_4_Cl, 15 mM MgCl_2_ and 5 mM β-mercaptoethanol (Figure 5).

### Assays for *in vitro* protein synthesis and frameshifting

Frameshifting assays were based on *in vitro* protein synthesis using an mRNA coding for the 27 kD ribosomal protein S2 that was modified to mimic the –1 frameshifting observed for the dnaX system (Supplementary Figure S1) (39,40). It used an S2 mRNA modified to carry an internal Shine-Dalgarno sequence (AGGGAG) at positions 365–370 and the slippery sequence GAAAAAAG at positions 381–388 of the coding sequence, as described (6), with the modifications that the slippery sequence was changed from AAAAAAAG to GAAAAAAG to eliminate low levels of a –2 frameshift product, and that no excess EF-G or EF-Tu were added to reaction mixtures. The effects of downstream mRNA secondary structure were simulated by hybridization of a DNA olgonucleotide with the sequence 5′-GTGCGCATCAGCGCTTCTTT-3′.

Following *in vitro* translation, [^35^S]-methioine-labeled products were separated and visualized by SDS-PAGE and autoradiography. Frameshifting levels were determined by quantification of bands corresponding to full-length and truncated products. Intensities were normalized based on the number of internal methionines in the predicted polypeptide products. Frameshifting was reported as the ratio of frameshifted product to full-length product.

## Results

### Structural evidence for intercalation of A1503

Intercalation of A1503 of 16S rRNA was first reported for the crystal structure of a ribosomal translocation intermediate complex trapped in the chimeric-hybrid state (3). A1503 was intercalated between bases –1 and –2 of the mRNA (where the first base of the P-site codon is defined as +1) (Figure 1). We asked whether this phenomenon was merely anecdotal, or more widespread among published x-ray and cryo-EM structures of ribosome complexes. We screened 672 deposited structures by calculating the distances between the respective centroids of the A1503 base and proximal mRNA bases, followed by visual inspection to identify 209 structures in which the relative positions of A1503 and nearby mRNA bases could be placed with reasonable confidence. This procedure identified 73 structures in which A1503 was intercalated, and an additional 89 where it was stacked on a single mRNA base; in 47 structures, A1503 was retracted out of range of intercalation or stacking.

**Figure 2.**
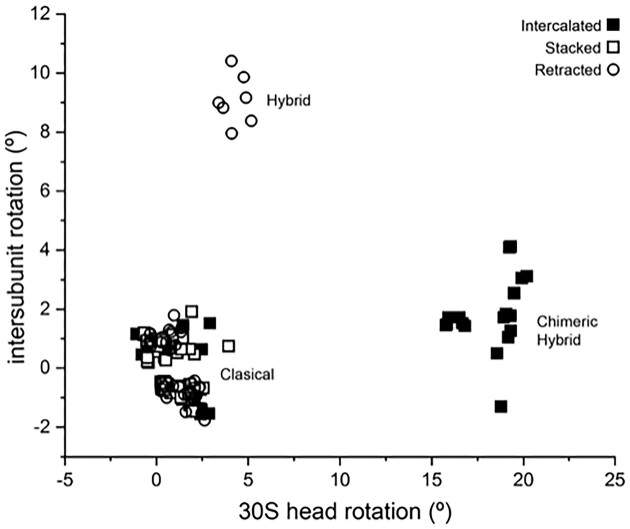
A1503 intercalation and ribosome rotational state. 30S head and body rotation values are plotted for 209 structures of ribosome complexes (Supplementary Table S1), relative to a classical-state reference structure (7K00). Symbols indicate whether A1503 is intercalated (▪), stacked on a single mRNA base (□) or retracted (○). Intercalation of A1503 is observed in all structures with rotation values corresponding to the chimeric-hybrid state, and in a subset of classical-state structures. No intercalation is observed in hybrid-state structures.

**Figure 3.**
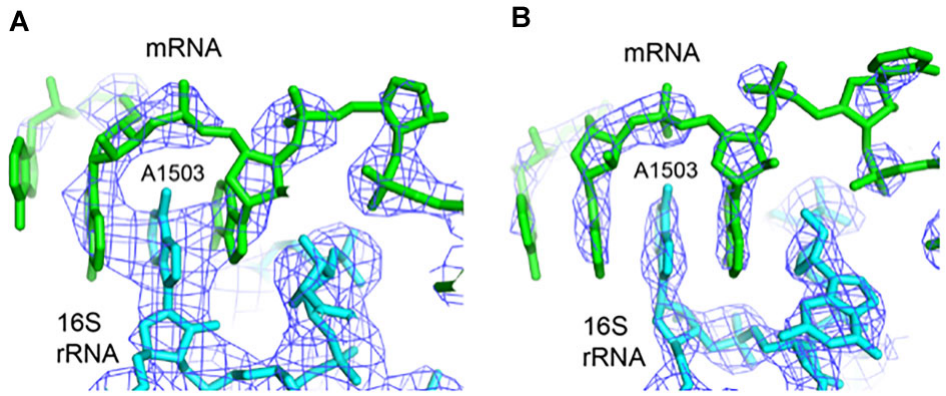
Examples of intercalation of A1503 in chimeric-hybrid and classical state ribosome structures. (**A**) 3.5Å resolution X-ray structure of a chimeric-hybrid state complex (PDB code 4V9K) (3). (**B**) 3.1 Å resolution X-ray structure of a classical state complex (PDB code 4V5P) (12).

**Figure 4.**
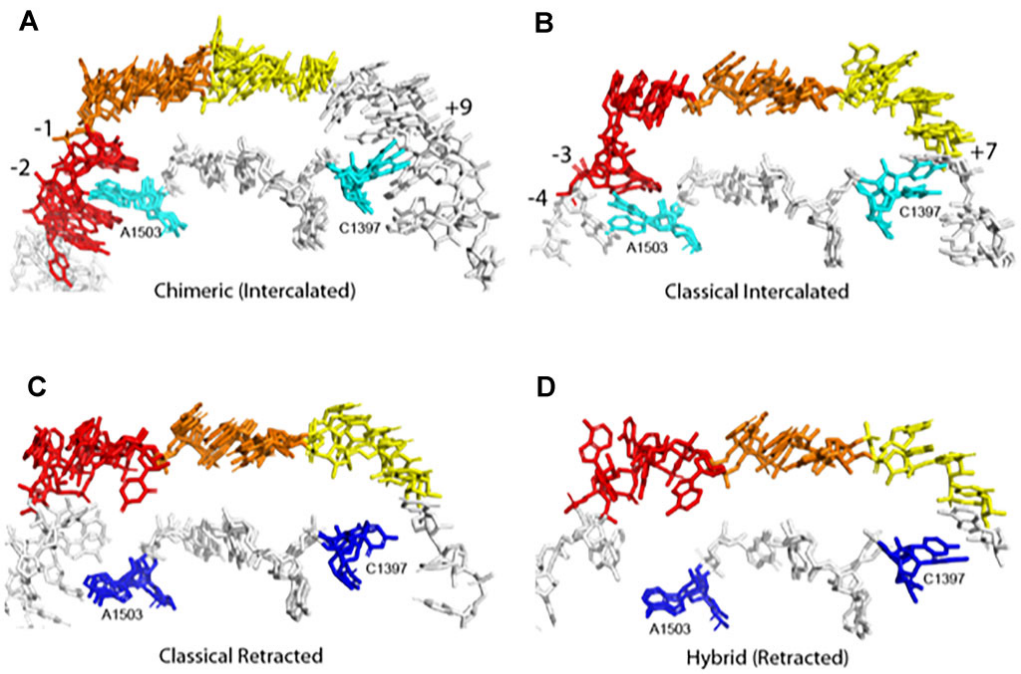
Positions of A1503 in different ribosomal states. Superimposition of multiple examples of ribosome complexes shows that A1503 is (**A**) intercalated between mRNA positions –1 and –2 in all chimeric-hybrid state complexes (examples from PDB codes 4W29, 4V9K, 4V9L, 6N1D); (**B**) intercalated between positions –3 and –4 (PDB codes 4WZD, 4WQ1, 4WZO) or (**C**) retracted (PDB codes 4V6F, 4V9D, 4V5F) in classical-state complexes and (**D**) retracted in all hybrid-state complexes (PDB codes 4V9D and 6WDG). The mRNA codons are colored according to A site (yellow), P site (orange) and E site (red). Note that stacking of C1397 on positions +7 or +9 is strongly correlated with intercalation of A1503.

We then asked whether intercalation of A1503 might be correlated with specific conformational states of the ribosome, assigned by their intersubunit and 30S head rotation values into classical (non-rotated), hybrid (∼6°–10° intersubunit rotation) or chimeric hybrid (∼15°–21° 30S head rotation) states (11). Measurement of the rotation angles of the 30S head and body domains for all of the above structures shows that those where A1503 is intercalated form two clusters, corresponding to the classical and chimeric-hybrid states (Figure 2). In all chimeric-hybrid structures, A1503 is intercalated. No intercalation was found in any hybrid-state complex. Examples of intercalation in chimeric-hybrid and classical state structures are shown in Figures 3 and 4.

In chimeric-hybrid state complexes, A1503 intercalates between bases –1 and –2, and in the classical-state structures between bases –3 and –4. This difference between the respective sites of intercalation can be explained by a 2-nucleotide shift of the mRNA relative to the 30S body domain in the chimeric-hybrid state (3,4). In the classical-state complexes, 55 show intercalation, 89 show stacking on a single mRNA base and 40 are retracted. The incidence of intercalation of A1503 is summarized in Table I; examples are shown in Figure 4.

### Constructing ribosomes with an abasic nucleotide at position 1503 of 16S rRNA

**Figure 5.**
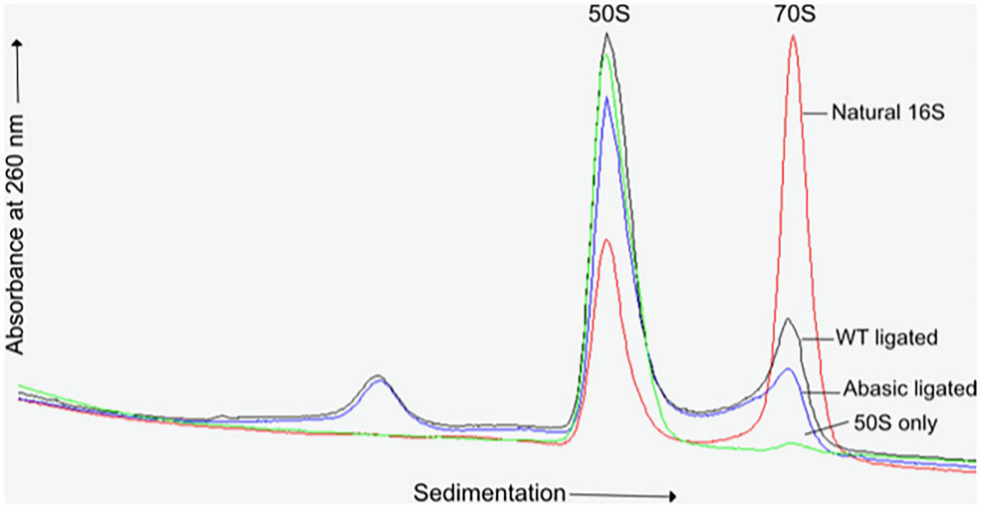
Isolation of reconstituted abasic ribosomes. 30S ribosomal subunits were reconstituted *in vitro* from semi-synthetic 16S rRNA constructed by ligation of the large 1491-nucleotide colicin fragment to a synthetic 49-mer RNA oligomer containing an abasic nucleotide at position 1503 or a wild-type (WT) sequence. Ribosomes were also reconstituted from natural full-length 16S rRNA for comparison. Reconstituted 30S subunits were associated with excess natural 50S subunits and the resulting 70S ribosomes were isolated by sucrose gradient centrifugation (Materials and methods).

Based on the above analysis and the previous proposal that intercalation of A1503 might act to prevent slippage of the translational reading frame during translocation of the mRNA (3), we sought to test this possibility by eliminating its intercalation. Since all four bases are capable of intercalation, we devised a scheme to abolish intercalation by creating ribosomes with an abasic nucleotide at position 1503 of 16S rRNA. This was done by replacing the 3′ 49-nucleotide section of the 1542-nucleotide 16S rRNA with a synthetic 49-mer containing an abasic nucleotide at position 1503. We took advantage of the ability of the colicin E3 endonuclease to make a single specific cut at position 1493, and ligated the resulting large 5′ 1493-mer fragment to a synthetic abasic 49-mer. This was accomplished by splinted ligation (13,14) using a novel strategy employing a tailed DNA splint which permits removal of the splint under mild conditions that preserve the native structure of the rRNA (Methods; Smart et al., unpublished). Abasic 70S ribosomes were then constructed from the semi-synthetic 16S rRNA by *in vitro* reconstitution with total 30S ribosomal proteins followed by association with natural 50S subunits (Figure 5; Materials and methods).

### Activity and frameshifting levels of abasic ribosomes

**Figure 6.**
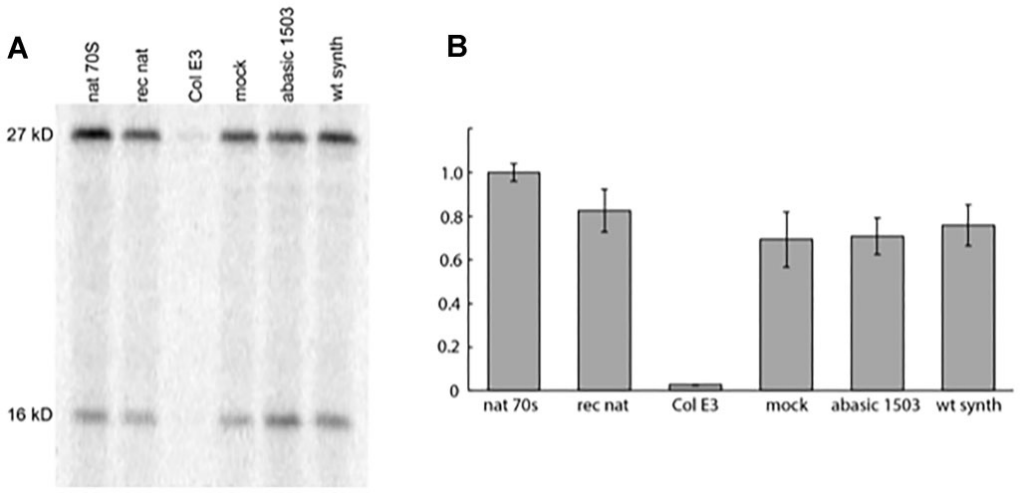
Semi-synthetic abasic ribosomes are active in *in vitro* protein synthesis. (**A**) Ribosomes reconstituted from semi-synthetic 16S rRNA containing an abasic nucleotide at position 1503 were tested for *in vitro* protein synthesis activity using a mRNA encoding the 27 kDa ribosomal protein S2 by incorporation of [^35^S]-methionine (6). The 16 kDa band corresponds to a premature termination product caused by a -1 frameshift at an engineered slippery sequence used in the experiments shown in Figures 7 and 8. (**B**) Protein synthesis activities of reconstituted ribosomes calculated as the sum of full-length and frameshifted products. Activities are presented as the fraction of that of wild-type natural tight-couple 70S ribosomes; standard errors of the mean are shown from N = 8 independent experiments (Table 2; Col E3 = 0.03 ± 0.00). Abbreviations indicate natural 70S ribosomes (nat 70S), and ribosomes reconstituted from natural 16S rRNA (nat rec), colicin E3 cleaved 16S rRNA (Col E3), natural 16S rRNA subjected to RNA ligation conditions (mock), and 16S rRNA constructed from ligation with the abasic 49mer (abasic 1503) or with a wild-type synthetic 49mer (wt synth).

We tested the activity of the reconstituted ribosomes in an *in vitro* assay that measures synthesis of a full-length 27 kD protein (6). Ribosomes reconstituted from natural 16S rRNA were 83% active compared to natural tight-couple ribosomes in translating a full-length protein; those constructed from ligated semi-synthetic wild-type 16S rRNA were 76% active, while ribosomes reconstituted from semi-synthetic 16S rRNA containing an abasic nucleotide at position 1503 were 71% active (Figure 6). We conclude that a base at position 1503 is not essential for protein synthesis activity *per se*, and that ribosomes reconstituted using 16S rRNA constructed by ligation to the synthetic 49mer have only modestly reduced activity relative to those reconstituted from natural 16S rRNA. Ribosomes reconstituted from colicin-cut 16S rRNA were inactive in protein synthesis.

**Figure 7.**
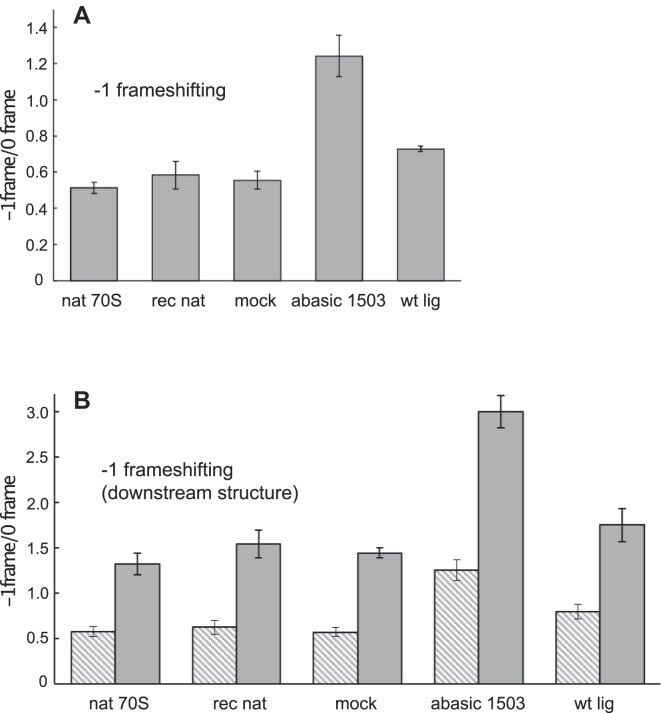
Frequencies of frameshifting *in vitro* (**A**) Increased –1 frameshifting by semi-synthetic ribosomes containing an abasic nucleotide at position 1503 (presented as the ratio of –1 frame to 0 frame translation) through an S2 mRNA containing a programmed frameshifting sequence. Frameshifting was quantified by the magnitude of the truncated 16 kDa polypeptide (Figure 6A) relative to that of full-length 27 kDa S2 protein (6). Data are from *N* = 8 independent experiments with mean and standard error values as reported in Table 2 (6). (**B**) The effects of the dnaX frameshift-stimulating downstream mRNA hairpin on –1 frameshifting was simulated by hybridizing a complementary 20-nucleotide DNA oligonucleotide 6 residues downstream from the slippery sequence (Materials and methods). Data from downstream mRNA hairpin simulating constructs were from *N* = 3 independent experiments (nat 70S = 1.32 ± 0.12, rec nat = 1.54 ± 0.15, mock = 1.44 ± 0.05, abasic 1503 = 3.00 ± 0.18, wt lig = 1.75 ± 0.18. Hatched bars in panel B show the data from panel A for comparison).

To measure frameshifting, we used an mRNA encoding ribosomal protein S2 that was previously designed to include a programmed frameshifting sequence based on the dnaX gene of *E. coli* (6) (Supplementary Figure S1). This sequence consisted of an internal Shine-Dalgarno-like sequence and a slippery A AAA AAG heptamer located 10 nucleotides downstream (15–17). Frameshifting into the -1 reading frame was measured by the magnitude of the [^35^S]-labeled 16kD premature termination product caused by the introduced programmed frameshifting sequence (6) (Figure 7A). Ribosomes reconstituted from natural 16S rRNA or mock-ligation procedures showed frameshifting levels similar to that of natural 70S ribosomes. However, semi-synthetic ribosomes containing an abasic nucleotide at position 1503 had a ratio of –1 to 0-frame product (–1/0 frame ratio) double that of wild-type ribosomes. Interestingly, ribosomes reconstituted from 16S rRNA constructed from ligation with a wild-type synthetic 49mer showed a small but significant increase in –1 frameshifting (‘wt lig’, Figure 7A; Table 2).

The wild-type dnaX-programmed frameshifting sequence includes a stimulatory hairpin element located 6 nucleotides downstream from the slippery heptamer (15), which is absent in our construct. We simulated the effect of this downstream secondary structure by hybridizing a complementary 20mer DNA oligonucleotide to a sequence 6 residues downstream from the mRNA slippery sequence (18). As expected, the downstream structure stimulated the levels of frameshifting for all ribosome constructs, and the abasic ribosomes showed frameshifting levels about double that of wild-type (Figure 7B). Again, the semi-synthetic wild-type ribosomes had a modestly elevated level of frameshifting relative to that reconstituted from natural 16S rRNA.

### Dimethylation of A1518 and A1519 confers a modest effect on maintaining the translational reading frame

**Figure 8.**
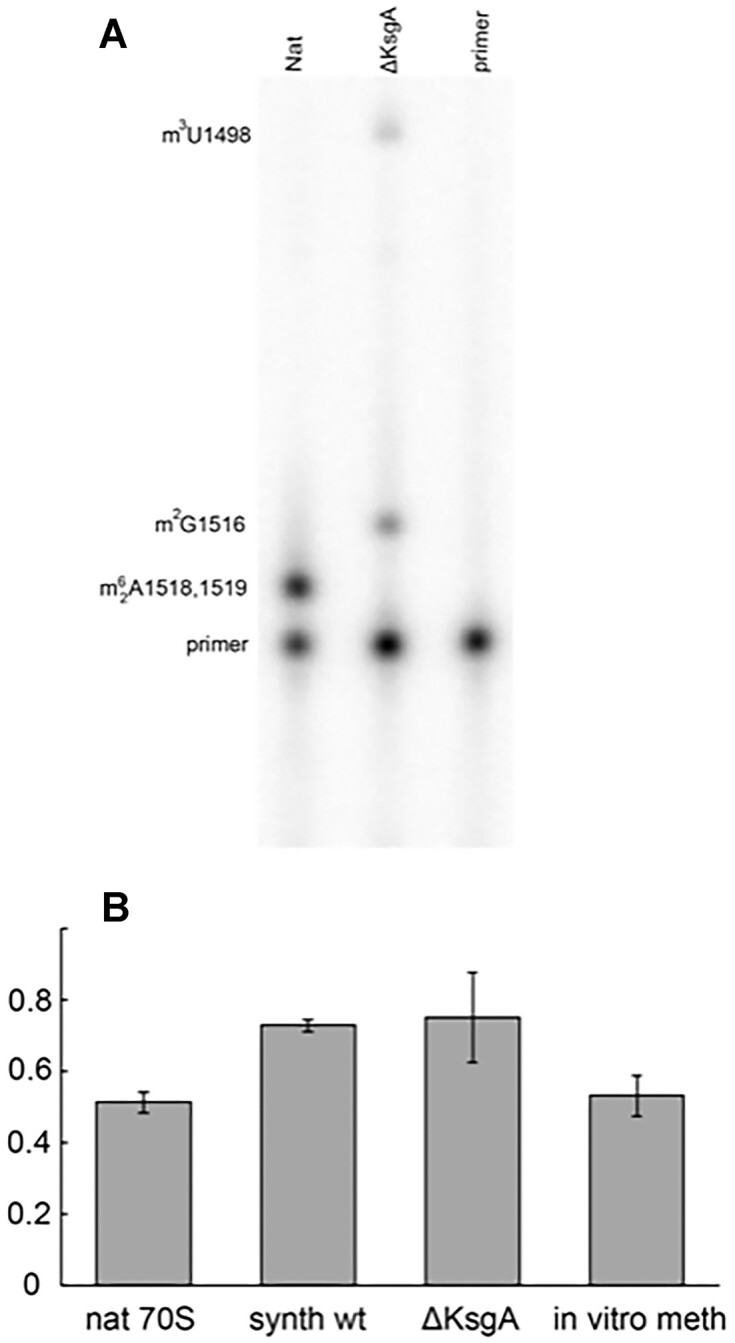
Increased frameshifting in non-methylated A1518-1519 ribosomes. (**A**) The *N*^6^_2_-dimethyladenosines at positions 1518 and 1519 cause a strong primer extension stop in wild-type natural 16S rRNA (Nat), which is absent in 16S rRNA from a methylase-deficient *KsgA* strain (ΔKsgA). (**B**) Semi-synthetic ribosomes containing the unmodified wild-type 49mer sequence (synth wt) or ribosomes purified from the methylase-deficient *KsgA* strain (ΔKsgA) have elevated levels of –1 frameshifting relative to natural 70S ribosomes (nat 70S), which are rescued by *in vitro* methylation (*in vitro* meth). Shown are mean values (Table 2) with error bars representing the standard error of the mean from independent experiments (*N* = 8 for nat 70S and synth wt); *N* = 5 for ΔKsgA and *in vitro* meth).

As noted above, a modest but reproducible increase in frameshifting was observed for semi-synthetic ribosomes constructed with the wild-type synthetic 49mer RNA (Figure 7). In natural 16S rRNA, this 49mer sequence contains the modified base *N*^6^,*N*^6^-dimethyladenine at positions 1518 and 1519, representing two of the most highly conserved post-transcriptional modifications in ribosomal RNA (19,20). We asked whether the absence of these modifications in the synthetic 49mer might contribute to the observed elevated level of frameshifting for the semi-synthetic wild-type ribosomes. To test this possibility, ribosomes with non-methylated 16S rRNA at positions A1518 and A1519 were purified from ribosomes from a *ksgA* deletion strain (ΔKsgA) (10), which lacks the dimethyl-A methylase. The absence of modifications at positions 1518 and 1519 was confirmed by primer extension of the 16S rRNA (Figure 8A).

Using the same assay as in Figure 7A, ribosomes isolated from the *ksgA* strain showed an increase in -1 frameshifting equivalent to that observed for the semi-synthetic ribosomes containing an intact A1503 (Figure 8B). Upon treatment of these ribosomes *in vitro* with purified KsgA methyltransferase, the level of frameshifting was rescued to a level that was indistinguishable from that of natural tight-couple ribosomes (Figure 8B). We conclude that the conserved methylations of A1518 and A1519 confer a modest but significant effect on preserving the translational reading frame. Thus, at least two elements in the 3′ 49 nucleotides of 16S rRNA contribute to reading frame accuracy.

## Discussion

Shifting of the translational reading frame generally leads to premature termination of an error-riddled polypeptide that is often toxic to the cell (1,21–23). Accordingly, it is not surprising that frameshifting error rates are typically about two orders of magnitude smaller than that for miscoding (1). Our understanding of how the translational machinery maintains the reading frame at such high accuracy in spite of the relative weakness of codon-anticodon base pairing (24,25) is still incomplete. Recent studies have implicated elongation factor EF-G in this process. During movement of the codon-anticodon duplex from the A site to the P site of the small subunit, pairing is no longer stabilized by the structure of the A site; during this vulnerable moment, structural studies have shown that the tip of domain IV of EF-G remains in contact with the minor groove of the duplex, apparently replacing the missing A-site contacts (2,3). Mutation of residues in the tip of domain IV causes frameshifting into –1 reading frame (5,6). Here, we ask whether the ribosome itself, and specifically its 16S ribosomal RNA, might play a role in maintaining the reading frame. This possibility was suggested by our previous structural observation that the highly conserved A1503 was intercalated between bases of the mRNA (3).

Our analysis of published structures of ribosome complexes shows that intercalation of the conserved A1503 into the mRNA is indeed widespread, and specific to certain conformational states of the ribosome (Figure 2). In some cases, A1503 stacks against one of the two mRNA bases at the intercalation site instead of full intercalation. A1503 intercalates between positions –1 and –2 in all examples of chimeric hybrid-state complexes, and between positions –3 and –4 in many classical-state structures (Table 1). We also note that intercalation or stacking of the conserved C1397 on the 5′ side of downstream base +10 in chimeric-hybrid complexes (or on +8 in classical-state complexes) is strongly correlated with intercalation of A1503. Both bases project from the tips of conserved, compact, tertiary hairpin-like structures (Figure 4). Moreover, the two hairpins are connected by a conserved, tertiary Watson-Crick base pair between C1399 and G1504, consistent with coupling of their movements during translocation. Both interactions would be expected to block slippage of the mRNA in either the forward or reverse direction.

**Table 1.** Intercalation of A1503 in mRNA^a^

State	Total	Interc	Positions	%	Stack	%	Retr	%
Classical	184	55	–3**|**−4	30	89	48	40	22
Hybrid	7	0	−	0	0	0	7	100
Chimeric Hybrid	18	18	−1**|**−2	100	0	0	0	0

^a^Summary of intercalation states of A1503 from 209 X-ray and cryo-EM structures, giving the numbers of structures in which A1503 is intercalated between two mRNA bases (Interc), stacked on a single mRNA base (Stack), or retracted out of range of intercalation (Retr), for the classical, hybrid and chimeric-hybrid states. PDB codes for all structures are listed in Supplementary Table S1. Positions of intercalation are indicated.

**Table 2.** Frameshifting levels^a^

70S construct	% –1 Frameshift	–1 Frameshift/0-Frame	Translation activity
Tight couple natural	33.8 ± 1.3	0.51 ± 0.03	1.00 ± 0.04
Natural 16S recon	36.8 ± 2.7	0.58 ± 0.08	0.83 ± 0.10
Mock ligated recon	35.7 ± 2.2	0.56 ± 0.05	0.69 ± 0.13
Abasic 1503 recon	55.4 ± 2.4	1.24 ± 0.11	0.71 ± 0.08
Ligated WT recon	42.1 ± 0.5	0.73 ± 0.01	0.76 ± 0.09
ΔKsgA submethylated	42.3 ± 2.7	0.75 ± 0.12	0.93 ± 0.01
Methylated *in vitro*	34.5 ± 1.7	0.53 ± 0.06	0.89 ± 0.09

^a^Efficiencies of –1 and +1 frameshifting and *in vitro* translation activities for each ribosomal construct (Figures 7 and 8). (% Frameshift) percent of product from –1 frameshift out of total product translated; (Frameshift/0-Frame) ratio of frameshifted product to 0-frame product. Recon indicates ribosomes constructed by *in vitro* reconstitution. Statistics are presented in the legends to Figures 6–8.

The position of nucleotide A1503 within the colicin E3 fragment near the 3′ end of 16S rRNA (9) suggested an experimental strategy to test the latter possibility. Using a novel splinted ligation technique, we replaced the 3′-terminal 49mer colicin fragment of 16S rRNA with a synthetic oligomer containing an abasic nucleotide at position 1503. Ribosomes reconstituted with the abasic rRNA show an increase in frameshifting into the –1 frame (Figure 7), providing support for a role for A1503 in maintaining the translational reading frame. We were unable to address the potential effects of stacking of base C1397 on the downstream mRNA, since C1397 lies outside the boundaries of the 49-nucleotide colicin E3 fragment.

We previously proposed that intercalation of A1503 could reversibly lock the register of the mRNA, preventing slippage of the translational reading frame at critical states during translocation, and retract to allow movement of the mRNA (3). A further possibility is suggested by our previous observation of -1 frameshifting during spontaneous translocation into the chimeric-hybrid state in the absence of EF-G (7). Intercalation of A1503 between positions –1 and –2 would interfere with base pairing of the P-tRNA anticodon with the adjacent base at position -1, preventing a shift into the –1 reading frame. In this way, A1503 could help establish a boundary for how far the P-tRNA can move during transition to the chimeric hybrid state simply by preventing the next two bases from pairing with tRNA.

Finally, in order for the mRNA to be translocated, A1503 must retract from its intercalated or stacked position. At what point in the series of rotational movements of the ribosome does this occur? The majority of classical-state and all chimeric-hybrid state ribosome structures in our analysis show intercalation of A1503, while no intercalation of A1503 was found in any hybrid-state complex (Figure 2). Since the first major rotational event in translocation involves forward rotation of the body of the 30S subunit to transition from the classical state to the hybrid state (26–29) we infer that retraction of A1503 must happen during this step. This conclusion is consistent with the observation that A1503 is intercalated at two different positions of the mRNA in the classical and chimeric-hybrid states (Figure 4). It is also compatible with the observation that no net translocational movement of the mRNA occurs during forward rotation of the small subunit body domain (29,30). Intercalation of A1503 in all chimeric-hybrid state structures in our analysis is in keeping with the apparent vulnerability of the ribosome to frameshifting during 30S head rotation (5–7).

We also observed a modest but significantly increased rlevel of frameshifting for ribosomes containing a wild-type synthetic 3′ 49mer (Figure 7). We tested the possibility that this could be due to the absence of the conserved methylations of A1519 and A1519 in the synthetic 49mer. Indeed, non-methylated ribosomes isolated from a methylase-deficient *ksgA* strain showed a similar increased level of frameshifting, which was reduced to wild-type levels by methylation *in vitro* using the ksgA methylase (Figure 8). Inspection of the interactions of nucleotides 1518 and 1519 with the surrounding ribosome structure does not suggest any obvious explanation for how their methylation could affect frameshifting; both bases are >8 Å from the nearest mRNA nucleotides and 20 Å from A1503. The effects of their methylation, albeit subtle, must therefore be indirect.

It is difficult to say how conserved our proposed mechanism would be, since there are relatively few structures available at sufficiently high resolution from diverse species. A1503 itself is highly conserved, although not universal (99.1%; *C* value of 1.926), according to our current alignment containing 1961 diverse sequences from bacteria, archaea and eukaryotes (41), suggesting that its intercalation is widespread.

The machinery of protein synthesis has evolved to stringently guard the accuracy of the translational reading frame (1). Previous studies have shown that elongation factor EF-G is an important player in this process (2–7,30). Our finding that elements of 16S rRNA also help to maintain the accuracy of the translational reading frame now reveal yet another functional role for ribosomal RNA in protein synthesis.

## Supplementary Material

gkae143_Supplemental_File

## Data Availability

The data underlying this article are available in the article and in its online supplementary material.

## Notes

Present address: Alexandria Smart, Helmholtz Institute for RNA-based Infection Research, 97080 Würzburg, Germany.
