# Supplementary material for: Implication of nucleotides near the 3′ end of 16S rRNA in guarding the translational reading frame
Source: Nucleic Acids Res. 2024 Mar 7;52(10):5950–8. doi: 10.1093/nar/gkae143 (PMC11162774; doi:10.1093/nar/gkae143)
Supplement: gkae143_Supplemental_File [file gkae143_supplemental_file.pdf]

Supplementary Information

Fig. S1. Complete sequence of the mRNA used for *in vitro* frameshifting experiments.

AUGCACCACCACCACCACCACGCAACUGUUUCCAUGCGCGACAUGCUCAAGGCU
GGUGUUCACUUCGGUCACCAGACCCGUUACUGGAACCCGAAAAUGAAGCCGUU
CAUCUUCGGUGCGCGUAACAAAGUUCACAUCAUCAACCUUGAGAAAACUGUACC
GAUGUUCAACGAAGCUCUGGCUGAACUGAACAAGAUUGCUUCUCGCAAAGGUAA
AAUCCUUUUCGUUGGUACUAAACGCGCUGCAAGCGAAGCGGUGAAAGACGCUG
CUCUGAGCUGCGACCAGUUCUUCGUGAACCAUCGCUGGCUGGGCGGUAUGCUG
ACUAACUGGAAAACCGUUCGUCAGUCCAUCAAACGUCUGAAAGACCUGGAAACU
CAGUCUCAGGGAGGUACUUUCGGAAAAAAGACCAAGAAAGAAGCGCUGAUGCGC
ACUCGUGAGCUGGAGAAACUGGAAAACAGCCUGGGCGGUAUCAAAGACAUGGG
CGGUCUGCCGGACGCUCUGUUUGUAAUCGAUGCUGACCACGAACACAUUGCUA
UCAAAGAAGCAAACAACCUGGGUAUUCCGGUAUUUGCUAUCGUUGAUACCAACU
CUGAUCCGGACGGUGUUGACUUCGUUAUCCCGGGUAACGACGACGCAAUCCGU
GCUGUGACCCUGUACCUGGGCGCUGUUGCUGCAACCGUACGUGAAGGCCGUUC
UCAGGAUCUGGCUUCCCAGGCGGAAGAAAGCUUCGUAGAAGCUGAGUAAGGAU
CCGAAUUCGAGCUCCGUCGACAAGCUUGCGGCCGCAGGACUCCGGAGGAGACU
CCGGAGUCCCUCGAG

Fig. S1. Complete sequence of the mRNA used for *in vitro* frameshifting experiments.

Highlight colors indicate Internal Shine-Dalgarno squence; Slippery sequence; -1 stop codon; 3' stem loop to create resistance to 3' exonucleases. AAAGAAGCGCUGAUGCGCAC indicates the sequence used to anneal the DNA oligonucleotide GTGCGCATCAGCGCTTCTTT to simulate downstream secondary structure.

Table S1. PDB Codes and 30S Subunit Head and Body Rotation Angles for Structures Identified in Figure 2.

A. Structures with A1503 Intercalated Into mRNA

PDB Code	Head Rotation	Body Rotation	Resolution (Å)	Method
6OPE-1	0.77	-0.69	3.1	X-RAY
6OSI-2	18.57	0.5	4.13	X-RAY
4WQ1-1	0.42	-0.74	3.1	X-RAY
4WR6-2	-0.5	0.95	3.05	X-RAY
4WRA-1	0.27	-0.66	3.05	X-RAY
4V51-1	-0.78	0.45	2.8	X-RAY
4V51-2	-1.06	1.15	2.8	X-RAY
4V5D-1	-0.52	0.97	3.5	X-RAY

4V5D-2	0.48	0.32	3.5	X-RAY
4V5L-1	-0.43	0.17	3.1	X-RAY
4V5P-1	1.01	0.94	3.1	X-RAY
4V5P-2	0.61	0.54	3.1	X-RAY
4V5Q-1	0.81	0.85	3.1	X-RAY
4V5Q-2	0.47	0.49	3.1	X-RAY
4V5R-1	0.4	0.55	3.1	X-RAY
4V5R-2	0.6	0.82	3.1	X-RAY
4V5S-1	0.69	0.75	3.1	X-RAY
4V5S-2	0.44	0.38	3.1	X-RAY
4V9J-1	19.31	4.11	3.86	X-RAY
4V9J-2	19.23	4.09	3.86	X-RAY
4V9K-1	16.51	1.72	3.5	X-RAY
4V9K-2	15.93	1.72	3.5	X-RAY
4V9L-1	16.68	1.52	3.5	X-RAY
4V9L-2	15.83	1.48	3.5	X-RAY
4V9M-1	16.82	1.43	4	X-RAY
4V9M-2	15.78	1.46	4	X-RAY
4W29-1	19.92	3.05	3.8	X-RAY
4W29-2	20.19	3.11	3.8	X-RAY
4W2E-1	2.87	-1.54	2.9	X-RAY
4W2F-1	-0.39	0.87	2.4	X-RAY
4W2F-2	0.24	-0.47	2.4	X-RAY
4W2G-1	-0.43	0.93	2.54	X-RAY
4W2G-2	0.32	-0.53	2.54	X-RAY
4W4G-2	2.42	-1.56	3.3	X-RAY
4WPO-1	0.3	0.65	2.8	X-RAY
4WQ1-2	-0.53	0.94	3.1	X-RAY
4WQF-1	1.46	1.46	2.8	X-RAY
4WQU-1	1.23	0.83	2.8	X-RAY
4WQY-1	1.01	0.83	2.8	X-RAY
4WU1-2	0.4	0.98	3.2	X-RAY
4WZD-1	1.94	-1.1	3.1	X-RAY
4WZD-2	0.47	0.95	3.1	X-RAY
4YPB-1	2.48	0.63	3.4	X-RAY
4YPB-2	2.24	-1.36	3.4	X-RAY
4YZV-2	2.46	-1.41	3.1	X-RAY
4Z3S-1	0.41	0.88	2.65	X-RAY
4Z3S-2	0.46	-0.52	2.65	X-RAY
4ZSN-2	2.39	-1.38	3.6	X-RAY
5DOY-1	-0.42	0.86	2.6	X-RAY
5DOY-2	0.74	-0.61	2.6	X-RAY
5E7K-2	0.38	0.99	3.2	X-RAY
5E81-2	-0.41	0.9	2.95	X-RAY
5EL4-2	0.6	1.04	3.15	X-RAY

5EL7-2	0.41	0.96	3.15	X-RAY
5J4B-1	-0.34	0.98	2.6	X-RAY
5J4C-1	0.32	0.96	2.8	X-RAY
5VPP-1	18.94	1.72	3.9	X-RAY
5VPP-2	19.18	1.05	3.9	X-RAY
5WIS-2	0.64	-0.59	2.7	X-RAY
5WIT-1	0.2	0.76	2.6	X-RAY
6GSK-2	0.58	0.99	3.36	X-RAY
6N1D-1	19.05	1.84	3.2	X-RAY
6N1D-2	19.5	2.54	3.2	X-RAY
6NWY-1	19.3	1.78	3.5	X-RAY
6NWY-2	19.31	1.26	3.5	X-RAY
6OF6-1	0.59	-0.7	3.2	X-RAY
6OJ2-2	0.27	0.77	3.2	X-RAY
6OPE-2	0.36	0.72	3.1	X-RAY
6OSI-1	18.78	-1.3	4.13	X-RAY
6OTR-2	2.17	-1.47	3.12	X-RAY
6OXI-2	1.82	-1.26	3.49	X-RAY
6QNQ-2	0.39	0.92	3.5	X-RAY
6UCQ-1	2.92	1.52	3.5	X-RAY

B. Structures with A1503 Stacked on mRNA

PDB Code	Head Rotation	Body Rotation	Resolution (Å)	Method
6OTR-1	2.09	0.47	3.12	X-RAY
6ORD-1	0.6	-0.54	3.1	X-RAY
6ORD-2	0.5	0.87	3.1	X-RAY
6OF6-2	0.22	0.9	3.2	X-RAY
6OJ2-1	1.2	-0.8	3.2	X-RAY
6O97-2	0.42	-0.45	2.75	X-RAY
6OF1-2	0.67	-0.67	2.8	X-RAY
6OXA-1	1.83	-0.55	3.25	X-RAY
6OXI-1	1.82	0.64	3.49	X-RAY
6UCQ-2	3.94	0.74	3.5	X-RAY
7JQM-2	1.95	-0.84	3.05	X-RAY
5W4K-2	0.48	-0.55	2.7	X-RAY
6CFJ-2	0.45	-0.57	2.8	X-RAY
6ND5-1	0.3	0.78	2.6	X-RAY
6ND5-2	1.05	-0.78	2.6	X-RAY
6ND6-2	0.75	-0.67	2.85	X-RAY
5J4B-2	0.49	-0.48	2.6	X-RAY
5J4C-2	0.43	-0.48	2.8	X-RAY
5VP2-2	0.62	-0.55	2.8	X-RAY
5EL5-2	-0.33	1	3.15	X-RAY
5EL6-2	0.59	1.03	3.1	X-RAY

5EL7-1	0.31	-0.72	3.15	X-RAY
4Y4P-2	0.79	-0.65	2.5	X-RAY
4YZV-1	2.41	-0.72	3.1	X-RAY
4ZSN-1	2.41	-0.73	3.6	X-RAY
4W2I-1	0.47	0.97	2.7	X-RAY
4W4G-1	2.56	-0.68	3.3	X-RAY
4V6G-2	0.27	0.87	3.5	X-RAY
4V5G-1	-0.1	0.54	3.6	X-RAY
4V5G-2	-0.48	0.22	3.6	X-RAY
1VY4-1	-0.26	0.85	2.6	X-RAY
1VY5-1	-0.37	0.78	2.54	X-RAY
1VY6-1	-0.45	0.83	2.9	X-RAY
1VY7-1	-0.42	0.78	2.8	X-RAY
4V63-1	1.44	-1.06	3.2	X-RAY
4V63-2	1.44	1.02	3.2	X-RAY
4V97-1	0.53	0.26	3.51	X-RAY
4W2I-2	0.26	-0.56	2.7	X-RAY
4WQR-1	0.76	-0.76	3.15	X-RAY
4WQR-2	-0.41	0.9	3.15	X-RAY
4WR6-1	0.79	-0.84	3.05	X-RAY
4WRA-2	-0.51	0.89	3.05	X-RAY
4WSD-1	0.96	-0.69	2.95	X-RAY
4WSD-2	0.54	0.93	2.95	X-RAY
4WT1-1	0.85	-0.74	3.05	X-RAY
4WT1-2	-0.5	0.95	3.05	X-RAY
4WU1-1	2.02	-0.97	3.2	X-RAY
4WZO-1	1.92	-0.94	3.3	X-RAY
4WZO-2	0.48	1.02	3.3	X-RAY
4Y4P-1	-0.3	0.84	2.5	X-RAY
5AFI-1	-0.47	0.34	2.9	CRYO-EM
5E7K-1	0.42	-0.72	3.2	X-RAY
5E81-1	0.45	-0.77	2.95	X-RAY
5EL4-1	1.79	-0.99	3.15	X-RAY
5EL5-1	1.03	-0.85	3.15	X-RAY
5EL6-1	0.41	-0.72	3.1	X-RAY
5IB7-1	0.9	-0.85	2.99	X-RAY
5IB8-1	1.68	-1.06	3.13	X-RAY
5IB8-2	0.49	0.91	3.13	X-RAY
5IBB-1	0.48	-0.75	2.96	X-RAY
5IBB-2	-0.43	0.87	2.96	X-RAY
5W4K-1	-0.34	0.89	2.7	X-RAY
5WIS-1	-0.29	0.81	2.7	X-RAY
5WIT-2	0.39	-0.53	2.6	X-RAY
6BUW-2	-0.66	1.12	3.5	X-RAY

6BZ6-2	-0.65	1.19	3.18	X-RAY
6CAE-1	0.54	0.84	2.6	X-RAY
6CAE-2	1.23	-0.61	2.6	X-RAY
6CFJ-1	-0.3	0.83	2.8	X-RAY
6GSK-1	1.34	-1	3.36	X-RAY
6GSL-2	-0.53	0.9	3.16	X-RAY
6N9F-1	0.68	0.93	3.7	X-RAY
6ND6-1	0.34	0.86	2.85	X-RAY
6NSH-2	0.9	1.12	3.39	X-RAY
6NUO-1	2.07	-0.76	3.2	X-RAY
6NUO-2	1.14	0.51	3.2	X-RAY
6O3M-1	2.09	-0.69	3.97	X-RAY
6O3M-2	1.37	0.63	3.97	X-RAY
6O97-1	-0.49	0.92	2.75	X-RAY
6OF1-1	0.33	0.9	2.8	X-RAY
6OXA-2	1.97	-1.46	3.25	X-RAY
6QNQ-1	1.76	-0.91	3.5	X-RAY
6UO1-1	0.42	0.85	2.95	X-RAY
6XQD-1	0.09	0.72	2.8	X-RAY
6XQE-1	0.34	0.94	3	X-RAY
7JQM-1	-0.49	0.93	3.05	X-RAY
7QV1-1	1.94	1.92	3.5	CRYO-EM
7QV3-1	1.94	1.92	5.14	CRYO-EM
7RQ9-2	0.52	-0.53	2.6	X-RAY

C. Structures with A1503 Retracted

PDB Code	Head Rotation	Body Rotation	Resolution (Å)	Method
7RQ8-1	-0.37	0.91	2.5	X-RAY
7RQ8-2	0.91	-0.62	2.5	X-RAY
7RQ9-1	-0.33	0.89	2.6	X-RAY
7JQL-1	-0.34	0.82	3	X-RAY
7JQL-2	1.9	-0.81	3	X-RAY
6XHV-1	-0.26	0.92	2.4	X-RAY
6XHV-2	0.44	-0.61	2.4	X-RAY
6XHW-1	0.25	0.87	2.5	X-RAY
6XHW-2	0.3	-0.53	2.5	X-RAY
6XHY-1	-0.29	0.84	2.6	X-RAY
6XHY-2	0.46	-0.65	2.6	X-RAY
7K53-1	2.17	-0.95	3.2	CRYO-EM

7LH5-1	0.29	-0.71	3.27	X-RAY
4V6G-1	1.85	-0.86	3.5	X-RAY
4V9D-2	1.34	1.22	3	X-RAY
5IB7-2	0.56	0.87	2.99	X-RAY
5VP2-1	-0.29	0.91	2.8	X-RAY
6NSH-1	2.34	-0.66	3.39	X-RAY
6OFX-1	0.96	1.8	3.3	CRYO-EM
6QNR-1	0.74	0.6	3.1	X-RAY
3JA1-1	5.18	8.38	3.6	CRYO-EM
4V4Z-1	2.63	-1.77	4.51	X-RAY
4V4J-1	1.61	-1.49	3.83	X-RAY
4V4Y-1	2.47	-1.53	5.5	X-RAY
4V5F-1	1.37	1.36	3.6	X-RAY
4V5F-2	1.04	0.78	3.6	X-RAY
4V5K-1	1.86	-0.99	3.2	X-RAY
4V6F-1	0.7	0.6	3.1	X-RAY
4V6F-2	-0.35	1.15	3.1	X-RAY
4V90-1	4.11	7.96	2.95	X-RAY
4V9D-1	3.4	8.99	3	X-RAY
5UQ7-1	1.91	-0.5	3.5	CRYO-EM
5UQ8-1	2.09	-0.43	3.2	CRYO-EM
6BUW-1	1.53	-0.9	3.5	X-RAY
6BY1-2	0.7	1.29	3.94	X-RAY
6BZ6-1	1.27	-0.65	3.18	X-RAY
6GSL-1	0.57	-1	3.16	X-RAY
6NTA-1	2.37	-0.64	3.1	X-RAY
6NTA-2	0.8	1.21	3.1	X-RAY
6QNR-2	-0.32	1.17	3.1	X-RAY
6YEF-1	2.08	-1.07	3.2	CRYO-EM
7N1P-1	-0.31	0.72	2.33	CRYO-EM
7SSL-1	4.76	9.86	3.8	CRYO-EM
7SSN-1	4.89	9.16	3.2	CRYO-EM
7SSO-1	3.65	8.82	3.2	CRYO-EM
7ST2-1	2.12	-0.93	2.9	CRYO-EM

7ST7-1	4.09	10.41	3.2	CRYO-EM
